# Real-World Outcomes of Ischemic Stroke Interventions in Older Patients: A Cohort Study from a Tertiary Medical Center

**DOI:** 10.3390/healthcare14131810

**Published:** 2026-06-23

**Authors:** Yochai Levy, Eisa Haj Ali, Estela Derazne, Dana Kagansky, Yichayaou Beloosesky, Miya Sharfman, Nadya Kagansky

**Affiliations:** 1 Shmuel Harofeh Geriatric Hospital, Beer Yaakov 7035001, Israel; 2 Gray Faculty of Medicine, Tel Aviv University, Tel Aviv 6997801, Israel; 3 Shamir (Assaf Harofeh) Medical Center, Beer Yaakov 7033001, Israel; 4 Adelson School of Medicine, Ariel University, Ariel 4070000, Israel

**Keywords:** acute ischemic stroke, reperfusion therapy, large vessel occlusion, geriatric patients, functional outcomes, real-world evidence

## Abstract

Background: Evidence regarding optimal reperfusion strategies in older patients with acute ischemic stroke remains inconsistent, particularly in real-world settings where patient selection and comorbidity burden differ from randomized trials. Objective: This study evaluates real-world outcomes of ischemic stroke interventions among older adults in a tertiary medical center. Methods: This single-center retrospective cohort study included patients aged ≥ 65 years with acute ischemic stroke admitted between August 2022 and December 2023. Mortality follow-up continued until September 2024. Patients were categorized according to treatment: intravenous thrombolysis (tPA) alone, endovascular thrombectomy (EVT) alone, or combined (tPA + EVT) therapy. Data included demographics, comorbidities, stroke severity (NIHSS), imaging, treatment timing, and mortality outcomes. Results: Among 200 patients (mean age 77.5 years), 66 received tPA, 86 underwent EVT, and 48 received tPA + EVT therapy. Patients treated with tPA alone presented with milder strokes and had low in-hospital and six-month mortality (1.5% and 6.1%, respectively). In contrast, EVT-only patients had substantially higher in-hospital and six-month mortality (21% and 43%, respectively). Patients receiving tPA + EVT therapy had low mortality rates comparable to the tPA group (2.1% and 6.3%, respectively), despite greater stroke severity at presentation. Conclusions: Despite greater stroke severity, patients receiving tPA + EVT therapy had outcomes comparable to those treated with tPA alone, while EVT-only patients experienced substantially higher mortality. These findings underscore the importance of patient selection and suggest that treatment allocation may strongly influence real-world outcomes in older adults.

## 1. Introduction

Stroke is a leading cause of mortality and disability worldwide and a major public health burden. In Israel, approximately 18,000 strokes occur annually, with ischemic stroke accounting for about 85% of cases [1,2,3,4]. However, only 40% of patients receive life-saving and disability-reducing treatment within the therapeutic time window [5]. The incidence of stroke increases markedly with age, with the majority of events occurring in older adults [6].

Currently, two main interventions exist for acute ischemic stroke: thrombolytic therapy using clot-dissolving drugs and endovascular thrombectomy (EVT). The most well-known thrombolytic drug is tissue plasminogen activator (tPA), which dissolves blood clots and is administered within 4.5 h of symptom onset, provided there are no contraindications. Although prior studies do not support restricting tPA based solely on age [7], conflicting evidence regarding its benefit in older patients, together with frequent contraindications, contribute to ongoing uncertainty reflected in current guidelines [8].

The primary concern is the risk of secondary hemorrhage, particularly when treatment is administered beyond the 3 h window from symptom onset. Due to this uncertainty and the limited available data, advanced age remains a common reason for withholding tPA therapy [9]. EVT is a minimally invasive procedure used to remove clots from large cerebral arteries in severe ischemic stroke cases. This procedure can be performed up to 24 h after stroke onset, with greater efficacy observed when performed earlier [2].

Large prospective studies have demonstrated that EVT significantly improves the recovery of patients with ischemic stroke caused by large vessel occlusion, with a higher proportion of patients achieving functional independence at 90 days compared to those receiving standard medical care alone [10,11,12]. The mortality rate in intervention groups in these studies was approximately 15%. Complications such as intracranial hemorrhage or 90-day mortality were similar or lower in EVT-treated groups, particularly when carefully selected patients were treated within controlled clinical trial settings. Real-world observational studies have reported similar outcomes in selected patients [13].

Based on these findings, current guidelines recommend intravenous thrombolysis for eligible patients without contraindications [8]. For suitable patients, EVT is recommended regardless of prior intravenous thrombolysis. Thus, many patients receive tPA + EVT therapy, while others receive a single intervention when eligibility criteria for one treatment are not met. However, despite these findings, real-world outcomes for EVT remain less consistent, with reports of worse outcomes in some settings [14,15], including interventions in older adults [16].

Population characteristics, healthcare system factors, and comprehensive clinical assessments may provide important insights into these treatment-related challenges.

Given the limited and sometimes conflicting evidence regarding reperfusion strategies in older adults, particularly in real-world clinical settings, further evaluation of treatment outcomes in routine practice is warranted. Intravenous thrombolysis and EVT represent the cornerstone reperfusion therapies for acute ischemic stroke, yet treatment allocation in routine clinical practice is often influenced by patient age, comorbidities, contraindications, stroke severity, and time from symptom onset. Consequently, outcomes observed in real-world settings may differ from those reported in randomized clinical trials.

This study examined the outcomes of ischemic stroke interventions among older adults treated at a tertiary medical center. The present study aimed to compare clinical outcomes among older adults receiving tPA alone, EVT alone, or tPA + EVT therapy. The primary independent variable was treatment strategy, while the primary outcomes were in-hospital and post-discharge mortality. We hypothesized that treatment outcomes in this older real-world population would differ from those reported in major clinical trials and that differences between reperfusion strategies would be observed.

## 2. Methods

This cohort study was conducted at a large tertiary hospital in Israel, where mechanical thrombectomy has been performed since 2017. Data were extracted from the electronic medical records of patients admitted between August 2022 and December 2023. Mortality follow-up continued for up to one-year post-hospitalization or until September 2024, whichever occurred first.

### 2.1. Patients

Patients aged 65 and older admitted for acute ischemic stroke and treated according to established guidelines [8,17] were included in the study. Patients who did not receive either intravenous thrombolysis (tPA) or EVT were excluded. All patients presenting to the emergency department with suspected stroke underwent urgent clinical and neurological evaluation by a neurologist, followed by computed tomography angiography (CTA) and CT perfusion imaging to guide treatment decisions. Patients diagnosed with acute ischemic stroke and meeting thrombolytic therapy criteria received tPA [7]. Patients who showed no rapid clinical improvement were referred for EVT, while those ineligible for tPA but meeting EVT criteria underwent EVT alone [12]. Patients were categorized into three groups based on the intervention received: tPA only, EVT only, or tPA + EVT therapy. The final number of participants included in each treatment group is presented in Section 3 and Table 1.

Clinical and demographic data were retrospectively extracted from the hospital electronic medical record system using a predefined data collection protocol. Collected variables included age, sex, ethnicity, comorbidities, medication use, functional and cognitive status, laboratory tests, stroke severity, imaging findings, and treatment-related time metrics. Stroke severity was assessed using the NIH Stroke Scale (NIHSS), a standard tool for evaluating stroke severity [18]. Additional data included times to hospital arrival and intervention, imaging results, occlusion locations, and blood test results. Most patients had documented baseline and discharge functional status based on their ability to perform basic activities of daily living (ADLs). Patients were categorized into the ADLs groups: completely independent, in need of supervision, minimal assistance (requiring minimal assistance with one to two basic ADLs), moderate assistance (requiring assistance with three to four basic ADLs), or maximal assistance (requiring assistance with all basic ADLs). The study was approved by the Institutional Review Board.

### 2.2. Outcomes

The primary independent variable was treatment allocation, categorized as intravenous thrombolysis alone (tPA), EVT, or tPA + EVT therapy. The primary outcomes were in-hospital mortality and post-discharge mortality during follow-up. Secondary outcomes included intracerebral hemorrhage, other bleeding complications, discharge destination, and post-discharge functional and cognitive status. Intracerebral hemorrhage (ICH) was defined as any hemorrhagic finding identified on follow-up brain CT imaging and documented in the medical record. Additional clinical and laboratory variables, including baseline patient characteristics, stroke severity, imaging findings, and admission laboratory parameters, were evaluated as potential factors associated with clinical outcomes.

### 2.3. Statistical Analysis

Sample size calculation was performed using the Select Statistics online calculator for comparing two proportions (https://select-statistics.co.uk/calculators/sample-size-calculator-two-proportions accessed on 20 June 2026)., based on the formula described by Wang and Chow [19].

Assuming an in-hospital mortality rate of 2% among tPA-treated patients and 12% among EVT-treated patients, with a two-sided alpha level of 0.05 and 80% power, a sample size of 99 patients per group was required. For six-month mortality, assuming rates of 6% and 25% in the tPA and EVT groups, respectively, a minimum of 54 patients per group was required.

A total of 200 patients were included in the analysis, comprising 66 patients treated with tPA alone, 86 with EVT alone, and 48 with tPA + EVT therapy.

Baseline demographic, clinical, laboratory, and imaging characteristics were compared between treatment groups using appropriate statistical tests according to variable type and distribution. Categorical variables were compared using the Chi-square test or Fisher’s exact test when appropriate. Continuous variables with normal distribution were analyzed using one-way analysis of variance (ANOVA), whereas non-normally distributed continuous and ordinal variables were analyzed using the Kruskal–Wallis test. Pairwise comparisons between the EVT and tPA + EVT groups were performed using the Chi-square test, Fisher’s exact test, or Mann–Whitney U test, as appropriate.

Survival analyses were conducted for three periods: during hospitalization, after hospital discharge, and across the entire follow-up period. Survival differences between treatment groups were assessed using Kaplan–Meier survival curves and compared using the log-rank test. Survival time was calculated from hospital admission (time zero) until death or censoring. Univariable and multivariable Cox proportional hazards regression analyses were performed to identify factors associated with mortality during follow-up. Age and sex were included in the multivariable model a priori. Albumin and C-reactive protein (CRP), which were statistically significant in univariate analysis, were also included. Although NIHSS was associated with mortality, it was strongly correlated with treatment allocation. Therefore, to better evaluate the independent impact of NIHSS on outcomes, it was entered into the multivariable model only after excluding the tPA-only treatment group, at which point its distribution was comparable between the EVT and the combined tPA + EVT groups. Statistical analysis was performed with IBM SPSS Statistics for Windows, version 29, Armonk, NY, USA: IBM Corp. A two-sided *p*-value ≤ 0.05 was considered statistically significant.

## 3. Results

Baseline characteristics of the patients are summarized in Table 1. A total of 200 patients were included in the analysis: 66 treated with tPA alone, 86 with EVT alone, and 48 with tPA + EVT therapy. Sex distribution was balanced, with 101 males and 99 females, and no significant differences between the groups. The mean patient age (±SD) was 77.5 ± 7.8 years, with no substantial differences among the groups. Patients in the EVT group had higher rates of atrial fibrillation (*p* < 0.001), were more frequently on anticoagulants (*p* < 0.001), and less commonly on antiplatelet therapy (*p* = 0.004). Aside from these differences (which reflect contraindications to tPA in anticoagulated patients), no significant differences were observed in other comorbidities, including diabetes mellitus, hypertension, or other chronic medication use, including cardiovascular risk factors, malignancies, or connective tissue diseases. Most patients were independent in ADLs across all groups. However, patients in the EVT group had slightly worse baseline functional status, a difference that reached statistical significance (*p* = 0.025).

Table 2 presents differences in stroke presentation. Time to intervention was significantly longer in the EVT group compared to the other groups. Among the 86 patients in the EVT group, 33 received treatment beyond the 4.5 h time window, whereas all patients in the other two groups were treated within 4.5 h (a significant difference, *p* < 0.001). Additionally, confusion was more prevalent among patients in the EVT group compared to the tPA + EVT group (36.9% vs. 17.0%; *p* = 0.018). Stroke severity, as assessed by the NIHSS, was milder in the tPA group compared to the other groups, with fewer cases of clinical signs such as hemiplegia, facial palsy, and aphasia. Imaging findings also differed: in the tPA group, not all patients exhibited clear occlusion, unlike the other groups, and fewer patients had large vessel occlusion. There were no significant differences in occlusion sites between the EVT and tPA + EVT groups. Discharge destination also differed between the groups, with a higher proportion of patients in the tPA group discharged home (73.8% compared to 35.5% in the EVT and 25.5% in the tPA + EVT groups; *p* < 0.001); accordingly, fewer were transferred to rehabilitation facilities compared to the other groups.

Table 3 shows laboratory findings at admission, highlighting significant differences among treatment groups. Hemoglobin levels were highest in the tPA group (median: 13.9 g/dL, IQR: 12.5–14.7), with a significant difference across groups (*p* = 0.002). Lymphocyte counts were also higher in the tPA group (1.9 × 10^9^/L, IQR: 1.4–2.4) compared to EVT (1.5 × 10^9^/L, IQR: 1.0–2.4) and tPA + EVT (1.5 × 10^9^/L, IQR: 1.0–2.2), *p* = 0.032. Similarly, albumin levels were notably elevated in tPA patients (4.0 g/dL, IQR: 3.7–4.1) compared to EVT (3.6 g/dL, IQR: 3.4–3.9) and tPA + EVT (3.7 g/dL, IQR: 3.5–3.9), *p* < 0.001. Inflammatory markers varied, with CRP levels significantly lower in the tPA group (2.0 mg/L, IQR: 1.0–5.0) compared to EVT (5.0 mg/L, IQR: 2.0–14.0) and tPA + EVT (3.5 mg/L, IQR: 1.0–8.0), *p* < 0.001. The neutrophil-to-lymphocyte ratio (NLR) was highest in the tPA + EVT group (4.1, IQR: 2.0–8.1) and showed a significant difference among groups (*p* = 0.035). The platelet-to-lymphocyte ratio (PLR) also differed significantly (*p* = 0.021), with the highest values observed in the EVT group (143.2, IQR: 97.5–226.7).

**Figure 1:**
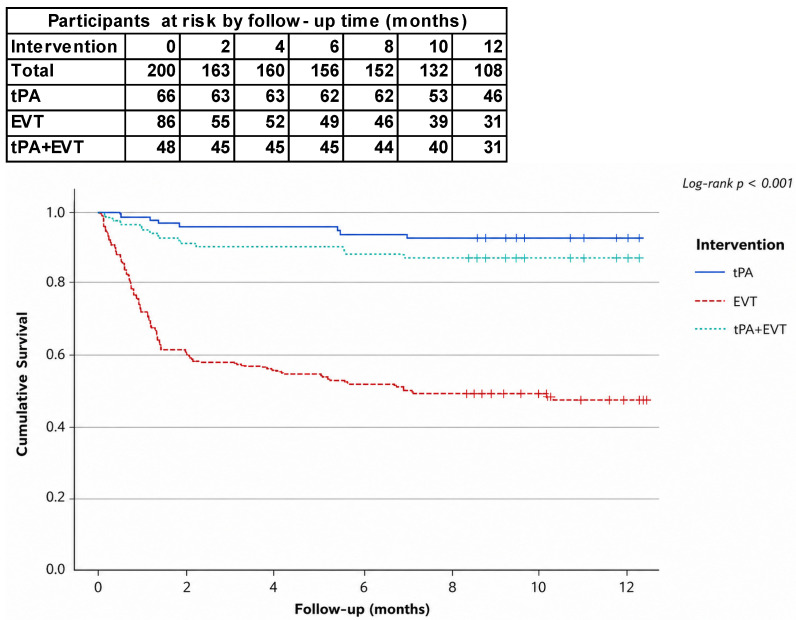
Kaplan–Meier survival curves showing cumulative survival over 12 months across treatment groups (tPA, EVT, and tPA + EVT). A significant difference in survival was observed between groups (log-rank test, *p* < 0.001).

Mortality outcomes are presented in Table 4 and illustrated in Kaplan–Meier survival curves (Figure 1). Mortality was significantly higher in the EVT group compared to the other two groups (*p* < 0.001). In-hospital mortality was 21%, and six-month mortality was 43% in the EVT group. By contrast, in the tPA group, in-hospital mortality was 1.5%, and six-month mortality was 6.1%. The tPA + EVT therapy group had similar mortality rates to the tPA group, with 2.1% in-hospital mortality and 6.3% six-month mortality. The difference in mortality between the EVT group and the other two groups remained consistent both during hospitalization and post-discharge, with no significant differences observed between the tPA and tPA + EVT therapy groups.

In Cox regression analyses (Table S1), EVT alone was associated with significantly higher mortality compared with tPA in both unadjusted and adjusted models (adjusted HR = 7.5, 95% CI 2.6–21.8; *p* < 0.001). The combined tPA + EVT group showed no significant difference from tPA in any model. When NIHSS was added to the model (restricted to EVT and tPA + EVT groups), EVT remained significantly associated with higher mortality (HR = 8.2, 95% CI 2.5–27.0; *p*< 0.001).

## 4. Discussion

This real-world cohort study examined reperfusion outcomes in older adults with acute ischemic stroke, with a mean age of 77.5 years. Patients in our cohort were substantially older than those enrolled in many landmark reperfusion trials, including the HERMES meta-analysis of EVT studies, in which the mean age was approximately 67 years [7]. This difference is clinically important, as older patients are underrepresented in clinical trials and often have a greater comorbidity burden, limiting the generalizability of trial findings to real-world populations.

The principal finding was an observed association with substantially higher mortality in patients treated with EVT alone compared with both the tPA + EVT therapy and tPA-alone groups, during hospitalization and in long-term follow-up. Notably, mortality rates in the tPA + EVT therapy group were comparable to those observed in the tPA-alone group, despite greater stroke severity at presentation. This observation may be clinically relevant when considering reperfusion strategies in carefully selected older patients.

Real-world data on EVT in older populations have shown inconsistent results. Subgroup analyses of older patients in RCTs such as SKIP [20] and SWIFT-DIRECT [21] did not identify significant differences between EVT-only and bridging therapy, while other real-world studies have suggested improved outcomes with tPA + EVT therapy [22,23,24]. The 43% mortality observed in the EVT-only group in the present study exceeds the approximately 15% EVT-related mortality reported in prior RCTs and meta-analyses [25,26,27,28], though it is consistent with findings in the older subgroups. For example, the MR CLEAN trial reported 90-day mortality of 50% in patients aged ≥80 years [29], and the HERMES meta-analysis reported 41% in those aged ≥85 years [16]. These discrepancies may reflect differences in patient selection and overall clinical complexity between randomized trial populations and real-world cohorts.

Several factors may partially explain the worse outcomes in the EVT-only group. Patients in this group more frequently presented outside the thrombolysis time window and exhibited higher inflammatory markers, including CRP and PLR, as well as lower albumin levels, biomarkers consistently associated with greater stroke severity, worse functional outcomes, and increased mortality [30,31,32]. Furthermore, as current guidelines favor tPA + EVT therapy when feasible, selection for EVT alone likely reflects underlying contraindications or reduced eligibility for tPA. Consistent with this interpretation, patients in the EVT-only group demonstrated significantly higher rates of atrial fibrillation and anticoagulant use, as well as longer times to intervention, factors that may have influenced thrombolysis eligibility and contributed to treatment selection bias.

In contrast to the EVT-only group, patients receiving tPA + EVT therapy presented with greater stroke severity than the tPA-alone group, yet demonstrated comparable mortality. This finding may reflect selective use of tPA + EVT therapy in patients otherwise deemed suitable for intervention despite more severe strokes. While this limits direct causal interpretation, it may support the consideration of tPA + EVT reperfusion strategies in appropriately selected older patients and underscores the importance of individualized assessment in a population characterized by substantial clinical heterogeneity.

### Limitations

The single-center retrospective design limits generalizability and the ability to fully account for all factors influencing treatment decisions, although the study was conducted in a high-volume center with established EVT experience [33]. Despite adjustment for selected variables, residual confounding may persist, and the observational nature of the study precludes causal inference. Functional outcomes, such as the modified Rankin Scale, were not consistently available, limiting assessment beyond mortality. While this restricts a comprehensive evaluation of treatment benefit, mortality remains a clinically meaningful and objective endpoint in this older, high-risk population, in whom survival itself represents a critical outcome.

## 5. Conclusions

In this real-world cohort of older stroke patients, EVT alone was associated with substantially higher observed mortality compared with tPA alone or tPA + EVT therapy. While differences in baseline severity and patient selection likely contribute to this finding, the favorable outcomes observed with thrombolysis either alone or in combination with EVT are consistent with its continued use as part of reperfusion strategies in appropriately selected older patients. These findings highlight the need for prospective studies focused specifically on older populations to define optimal treatment strategies, refine patient selection criteria, and address the persistent gap between trial evidence and real-world practice in this growing and underserved group.

## Figures and Tables

**Table 1:** Baseline Characteristics of Patients by Treatment Group.

	Intervention						*p*-Value
	tPA		EVT		tPA + EVT		
Age, Mean ± SD	76.9 ± 8.4		77.9 ± 7.3		77.5 ± 7.7		0.713
	*N*	%	*N*	%	*N*	%	
Total	66	100.0	86	100.0	48	100.0	0.141
Men	39	59.1	37	43.0	25	52.1	
Women	27	40.9	49	57.0	23	47.9	
Previous CVA_ TIA	13	19.7	22	25.6	6	12.5	0.309
Cognitive impairment	6	9.2	13	15.7	3	6.4	0.224
Atrial Fibrillation	4	6.1	42	48.8	10	20.8	<0.001 *
Hypertension	60	90.9	84	97.7	45	93.8	0.187
Hyperlipidemia	57	86.4	77	89.5	43	89.6	0.802
Diabetes mellitus	30	45.5	35	40.7	16	33.3	0.428
History of IHD	15	22.7	28	33.3	17	35.4	0.252
Carotid stenosis							0.388
Unknown	53	80.3	72	84.7	36	75.0	
<50%	5	7.6	8	9.4	6	12.5	
50–70%	6	9.1	1	1.2	3	6.3	
>70%	2	3.0	4	4.7	3	6.3	
CKD	11	16.7	10	11.6	4	8.3	0.393
Malignancy	13	19.7	19	22.1	9	18.8	0.882
Obesity	4	6.1	5	5.8	4	8.3	0.833
Smoking	14	21.2	13	15.1	6	12.5	0.419
Antihypertension treatment	55	83.3	77	91.7	39	83.0	0.220
Hyperlipidemia treatment	44	66.7	65	77.4	38	80.9	0.173
Antiplatelet therapy							0.004 *
Aspirin	33	50.8	18	22.5	20	44.4	
Clopidogrel	4	6.2	6	7.5	5	11.1	
No Treatment	28	43.1	56	70.0	20	44.4	
Anticoagulation therapy	2	3.0	35	41.2	5	10.6	<0.001 *
Insulin therapy	9	13.6	11	13.1	2	4.3	0.225
Baseline functional status							0.025 *
Maximal assistance	1	1.5	1	1.2	1	2.1	
Moderate assistance	0	0.0	4	4.7	0	0.0	
Minimal assistance	1	1.5	6	7.0	0	0.0	
Supervision	3	4.5	1	1.2	1	2.1	
Modified independence	13	19.7	22	25.6	7	14.6	
Complete independence	48	72.7	52	60.5	39	81.3	

* *p* < 0.05. Abbreviations: tPA, tissue plasminogen activator; EVT, endovascular thrombectomy; SD, standard deviation; CVA, cerebrovascular accident; TIA, transient ischemic attack; IHD, ischemic heart disease; CKD, chronic kidney disease; N, number.

**Table 2:** Presentation characteristics and in-hospital outcomes by treatment group.

	Intervention						
	tPA		EVT		tPA + EVT		*p*-Value
	*N*	%	*N*	%	*N*	%	
Presentation to intervention							<0.001 *
<4.5 h	66	100.0	53	61.6	48	100.0	
4.5–6 h	0	0.0	28	32.6	0	0.0	
>6 h	0	0.0	5	5.8	0	0.0	
Door to intervention							<0.001 *
within 1 h	64	97.0	44	51.2	48	100.0	
after 1	2	3.0	40	46.5	0	0.0	
after more than 1 h	0	0.0	2	2.3	0	0.0	
Occlusion	43	65.2	86	100.0	48	100.0	<0.001 *
Occlusion Intracranial ICA	3	4.5	13	15.1	8	16.7	0.072
Occlusion M1-MCA	11	16.7	43	50.0	25	52.1	<0.001 *
Occlusion M2-MCA	13	19.7	22	25.6	13	27.1	0.595
Occlusion PCA	1	1.5	0	0.0	2	4.2	0.164
Occlusion ACA	3	4.5	5	5.8	0	0.0	0.248
Occlusion Basilar	2	3.0	5	5.8	4	8.3	0.465
Occlusion Other	11	16.7	3	3.5	1	2.1	0.002 *
NIHSS							<0.001 *
<5	34	52.3	4	4.9	3	6.8	
5–15	29	44.6	59	72.0	29	65.9	
16–20	2	3.1	19	23.2	12	27.3	
21–42	0	0.0	0	0.0	0	0.0	
Confusion	5	7.6	31	36.9	8	17.0	<0.001
No complications	63	95.5	58	69.0	33	70.2	<0.001 *
ICH	3	4.5	26	31.0	13	27.7	
Hemorrhage other	0	0.0	0	0.0	1	2.1	
Discharge destination							
In hospital mortality	1		18		1		
Home	48	73.8	24	35.3	12	25.5	<0.001 *
Nursing home	1	1.5	11	16.2	5	10.6	
Inpatient Rehabilitation	15	23.1	33	48.5	30	63.8	
Other hospital	1	1.5	0	0.0	0	0.0	

* *p* < 0.05. Abbreviations: tPA, tissue plasminogen activator; EVT, endovascular thrombectomy; ICA, internal carotid artery; MCA, middle cerebral artery; PCA, posterior cerebral artery; ACA, anterior cerebral artery; NIHSS, National Institutes of Health Stroke Scale; ICH, intracerebral hemorrhage.

**Table 3:** Admission blood test results by treatment group.

	Intervention									*p*-Value
	tPA			EVT			tPA + EVT			
	*N*	Median	Median (IQR)	*N*	Median	Median (IQR)	*N*	Median	Median (IQR)	
HGB g/dL	66	13.9	12.5–14.7	86	12.6	11.4–13.9	48	13.0	11.9–14.1	0.002 *
NEU ×10^9^/L	65	5.4	3.8–7.3	86	6.0	4.7–8.1	48	5.4	4.4–7.7	0.266
LYM × 10^9^/L	66	1.9	1.4–2.4	86	1.5	1.0–2.4	48	1.5	1.0–2.2	0.034 *
PLT × 10^9^/L	65	211.0	179–252	86	219.5	176–267	48	194.0	170–235	0.079
INR	64	1.0	1–1	86	1.0	1.0–1.1	48	1.0	1–1	0.136
PT sec	65	11.0	11–12	85	12.0	11–13	48	12.0	11–13	0.005 *
PTT sec	65	29.0	27–31	85	28.0	26–31	48	28.0	26.0–29.5	0.351
CRP mg/L	63	2.0	1–5	85	5.0	2–14	48	3.5	1–8	0.001 *
RDW %	66	14.0	13–15	86	14.0	13–15	48	14.0	13–14	0.121
MPV fL	66	8.0	8–9	86	9.0	8–9	48	8.0	8–9	0.406
HDL mg/dL	38	43.0	34–54	49	42.0	34–49	33	43.0	36–52	0.756
LDL mg/dL	37	89.0	60–111	48	75.0	59–105	33	76.0	66–99	0.645
Albumin g/dL	61	4.0	3.7–4.1	84	3.6	3.4–3.9	48	3.7	3.5–3.9	<0.001 *
TSH µIU/mL	34	1.7	1.1–2.9	32	1.8	0.9–3.0	23	1.5	0.9–3.1	0.941
NLR	65	2.8	1.9–4.1	86	3.9	2.5–6.7	48	4.1	2.0–8.1	0.035 *
PLR	65	115.9	83.6–155.0	86	143.2	97.5–226.7	48	124.3	86.3–212.4	0.021 *

* *p* < 0.05. Abbreviations: tPA, tissue plasminogen activator; EVT, endovascular thrombectomy; HGB, hemoglobin; PLT, platelet count; INR, international normalized ratio; PT, prothrombin time; PTT, partial thromboplastin time; CRP, C-reactive protein; RDW, red cell distribution width; MPV, mean platelet volume; HDL, high-density lipoprotein; LDL, low-density lipoprotein; TSH, thyroid-stimulating hormone; NLR, neutrophil-to-lymphocyte ratio; PLR, platelet-to-lymphocyte ratio.

**Table 4:** In-hospital and 6-month mortality by treatment group.

	Intervention						*p*-Value	* *p*-Value	# *p*-Value
	tPA N = 66		EVT N = 86		tPA + EVT N = 48				
Mortality	*N*	% (95% CI)	*N*	% (95% CI)	*N*	% (95% CI)			
In-hospital	1	1.5 (0.04–8.2)	18	20.9 (12.9–31.1)	1	2.1 (0.05–11.1)	<0.001	<0.001	0.995
6 Months	4	6.1 (1.7–14.8)	37	43.0 (32.4–54.2)	3	6.3 (1.3–17.2)	<0.001	<0.001	1.000

Abbreviations: tPA, tissue plasminogen activator; EVT, endovascular thrombectomy; N, number of patients; %, percentage. *p*-value, EVT vs. tPA; * *p*-value, EVT vs. tPA + EVT; # *p*-value, tPA vs. tPA + EVT.

## Data Availability

The data presented in this study are available on request from the corresponding author. The data are not publicly available due to ethical restrictions.

## Supplementary Materials

The following supporting information can be downloaded at: https://www.mdpi.com/article/10.3390/healthcare14131810/s1, Table S1: Association between intervention and mortality during all study period.
