# Supplementary material for: Real-World Outcomes of Ischemic Stroke Interventions in Older Patients: A Cohort Study from a Tertiary Medical Center
Source: Healthcare (Basel). 2026 Jun 23;14(13):1810. doi: 10.3390/healthcare14131810 (PMC13362340; doi:10.3390/healthcare14131810)
Supplement: Supplementary file 1 [file healthcare-14-01810-s001.zip › healthcare-4314271-supplementary.pdf]

Table S1. Association between Intervention and mortality during all study period. Results from Cox regression models

	Intervention		
**Model**	**tPA**	**EVT**	**tPA+EVT**
**Unadjusted**			
Cases	4	42	4
Follow-up (months)	723	591	519
Crude Rate/1000 months	5.5	71.1	7.7
HR	1	10.7	1.4
95% CI		3.8–29.8	0.3–5.5
P		<0.001	0.645
**Adjusted** *			
Cases	4	40	4
Follow-up (months)	637	567	519
Crude Rate/1000 months	6.3	70.6	7.7
HR	1	7.5	1.2
95% CI		2.6–21.8	0.3–4.8
P		<0.001	0.824
**Adjusted** **			
Cases	-	38	3
Follow-up (months)	-	541	484
Crude Rate/1000 months	-	70.2	6.2
HR	-	8.2	1
95% CI	-	2.5–27.0	
P	-	<0.001	
**5 ≤ NIHSS ≤ 15**			
Cases	2	28	2
Follow-up (months)	265.3	389.0	324.2
Crude Rate/1000 months	7.5	72.0	6.2
HR	1	6.3	0.8
95% CI		1.4–27.5	0.1–0.5
P		0.015	0.787

Abbreviations: tPA, tissue plasminogen activator; EVT, endovascular thrombectomy; HR, hazard ratio; CI, confidence interval; NIHSS, National Institutes of Health Stroke Scale.

HR - Hazard Ratio

95% CI - 95% confidence interval for Hazard Ratio

*Adjusted for age, sex, albumin and CRP

** Adjusted for age, sex, albumin, CRP and NIHSS
